# Relationship between visceral obesity and prognosis in patients with stage IVB cervical cancer receiving radiotherapy and chemotherapy^☆^

**DOI:** 10.1016/j.cpt.2023.09.002

**Published:** 2023-09-20

**Authors:** Chao Ji, Silin Liu, Che Wang, Jie Chen, Jin Wang, Xinyue Zhang, Jinlu Ma, Mengjiao Cai

**Affiliations:** Department of Radiation Oncology, The First Affiliated Hospital of Xi'an Jiaotong University, Xi'an, Shaanxi 710061, China

**Keywords:** Cervical cancer, Chemoradiotherapy, Visceral obesity, Prognosis

## Abstract

**Background:**

Concurrent chemoradiotherapy is the preferred treatment for stage IVB cervical cancer; however, some patients experience a poor prognosis. The prognostic significance of body composition indicators, including visceral obesity, has been extensively investigated in patients with cancer. This study aimed to assess the impact of body composition indicators, specifically pretreatment fat content, on the survival outcomes of patients with stage IVB cervical cancer.

**Methods:**

We retrospectively analyzed clinical information from patients diagnosed with stage IVB cervical cancer between 2010 and 2018. We measured visceral obesity (visceral-to-subcutaneous adipose tissue area ratio [VSR]) and skeletal muscle index (SMI) on pretreatment computed tomography (CT) images. We evaluated the impact of these body composition parameters on the prognosis of patients with cervical cancer.

**Results:**

Overall, 116 patients were included, 81 of whom had complete clinical and imaging information. Based on the cut-off values from X-tile analysis, we categorized patients into high and low VSR and SMI groups. The overall survival (OS) rate of patients with a high VSR was significantly higher than that of patients with a low VSR (*P* = 0.022). Multivariate Cox regression analysis showed that a low VSR was an independent risk factor for the prognosis of patients with stage IVB cervical cancer.

**Conclusion:**

Visceral obesity before radiotherapy and chemotherapy has a protective effect on the prognosis of patients with stage IVB cervical cancer, while low muscle index and VSR are associated with poor prognosis.

## Highlights

• In patients with stage IVB cervical cancer, the overall survival (OS) rate of patients with high visceral-to-subcutaneous adipose tissue area ratio (VSR) is significantly higher than those with low VSR.
• A low VSR is an independent risk factor for poor prognosis in patients with stage IVB cervical cancer.
• Visceral obesity before chemoradiotherapy has a protective effect on the prognosis of patients with stage IVB cervical cancer.

## Graphical abstract

CI: Confidence interval; SMI: Skeletal muscle index; VSR: Visceral/subcutaneous fat area ratio.

**Figure:**
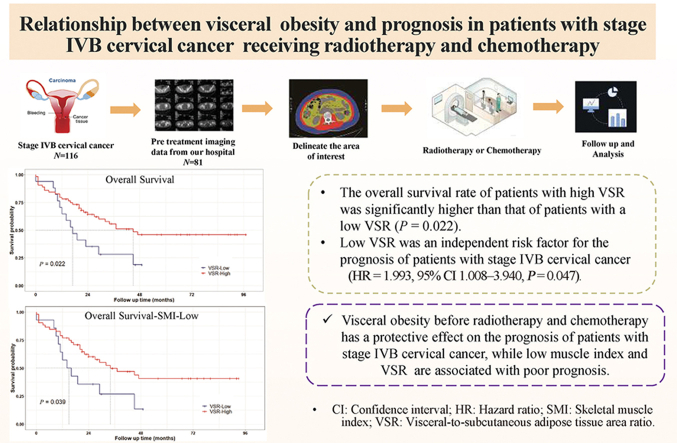

## Introduction

Cervical cancer is the fourth most common cancer in women worldwide. In recent years, the incidence and mortality rates of cervical cancer in China have been increasing annually. This trend is particularly evident among the youth,^1^ posing a serious threat to women's health in China and imposing a significant economic burden on society. According to National Comprehensive Cancer Network (NCCN) guidelines, the preferred treatment option for patients with stage IVB cervical cancer is systemic comprehensive treatment with concurrent chemoradiotherapy. However, during this treatment, patients may experience gastrointestinal toxicity, leading to weight loss and malnutrition,^2^ resulting in muscle tissue depletion, changes in body composition, and functional alterations, affecting prognosis.^3^ Identifying prognostic markers based on patients' general physical and nutritional status is paramount to improving patients' tolerance to radiotherapy and chemotherapy, improving the prognosis and quality of life of patients with advanced cancer, and maximizing treatment effectiveness.

Body composition assessment usually includes the quantification of fat and muscle mass. Research has shown associations between visceral obesity, sarcopenia, sarcopenic obesity, and the prognosis of various cancer types^4^, ^5^, ^6^ Visceral obesity refers to the excessive accumulation of visceral fat in the abdominal cavity,^7^ calculated as the ratio of the visceral fat area (VFA) to the subcutaneous fat area (SFA). High VSR is defined as visceral obesity.^8^^,^^9^ Computed tomography (CT) is an important method for tumor imaging. Researchers have discovered that measuring the area of individual skeletal muscle and adipose tissue on a CT scan cross-sectional image at the third lumbar vertebral body (L3) level can effectively reflect the quantity of muscle and adipose tissue throughout the body^10^^,^^11^ This implies that body composition data obtained through CT imaging can serve as novel prognostic markers for diseases. Previous studies have shown that visceral and sarcopenic visceral obesity is associated with poor prognosis after pancreatic cancer resection.^12^ Kim et al. found that a larger SFA is associated with better disease-free survival in patients with colon cancer.^13^ However, the impact of body composition indicators, such as visceral obesity, on patients with locally advanced cervical cancer remains unclear.

Therefore, this study collected relevant indicators such as VSR, VFA, SFA, and skeletal muscle index (SMI) of patients with stage IVB cervical cancer to analyze their levels of visceral obesity, muscle mass, and other physical conditions. This study investigated the impact of body composition parameters and a combination of these factors on the prognosis of patients with stage IVB cervical cancer.

## Methods

### Patients

We gathered 116 patients diagnosed with stage IVB cervical cancer according to the 2009 International Federation of Gynecology and Obstetrics (FIGO) classification. These patients underwent radical radiotherapy or chemotherapy at the First Affiliated Hospital of Xi'an Jiaotong University between December 2010 and October 2018. The diagnoses were confirmed through biopsy and imaging. For subsequent analysis, we screened 81 patients from our hospital who possessed CT imaging data before treatment. The CT scans had a slice thickness of ≤5 mm. Clinical information related to the patient's age, body mass index (BMI), disease stage, and treatment method was obtained from their medical records.

### Image analysis

**Figure 1:**
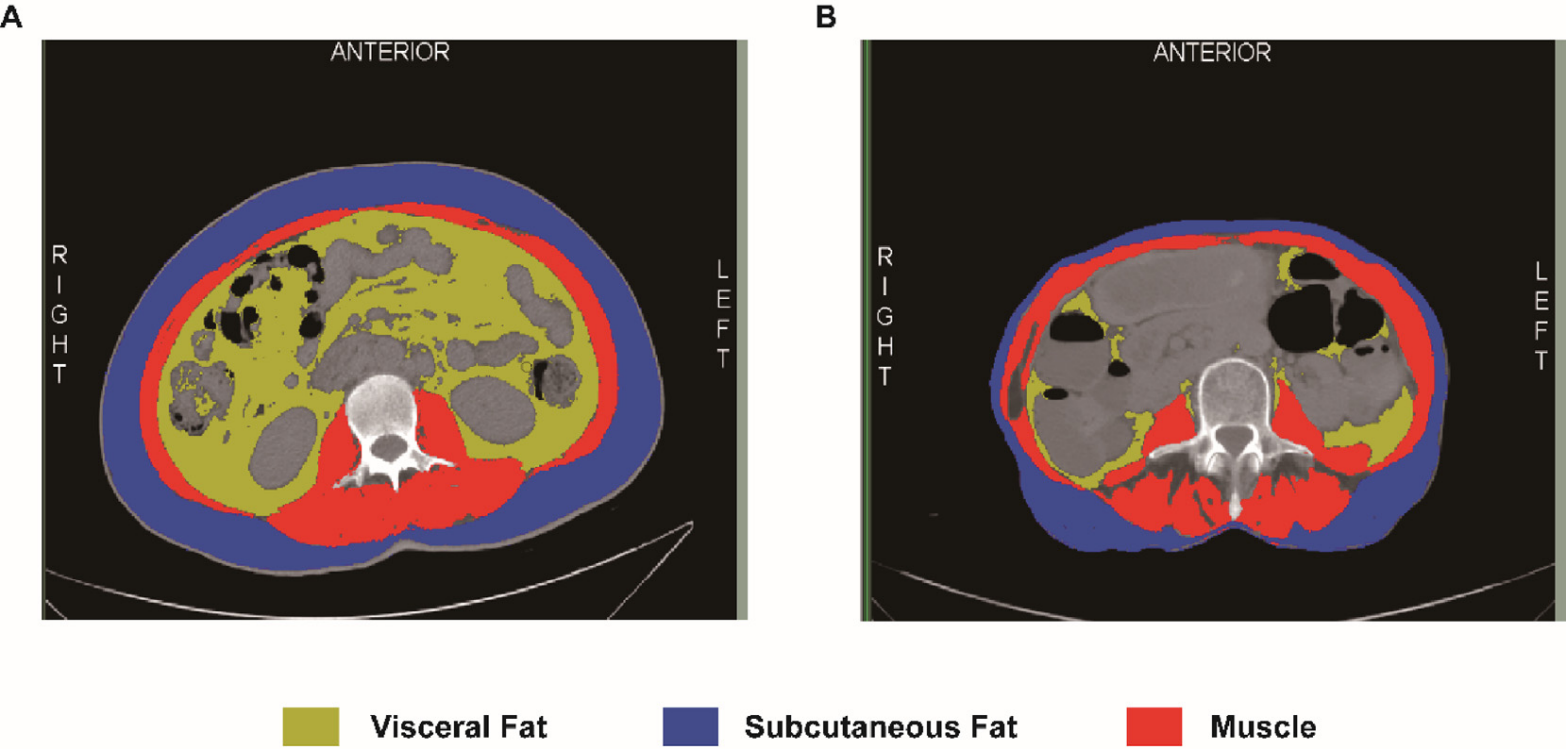
Body composition measurement of L3 horizontal abdominal CT cross-section. (A and B) Cross-sectional CT images were taken at the level of the third lumbar vertebra. The yellow area represents visceral fat, with an HU threshold of −150 to −50 HU; The blue area represents subcutaneous fat, with an HU threshold of −190 to −30 HU; The red area represents total skeletal muscle, with an HU threshold of −29 to 150 HU. CT: Computed tomography; HU: Huntsfield unit.

A certified and experienced radiologist used Slice-O-matic version 5.0 revision 7 (Tomovision, Montreal, Canada), user-friendly software known for its excellent technical support,^14^ to analyze abdominal CT images at the third lumbar vertebra level. This analysis was conducted without knowing the patient's information. The aim was to determine the cross-sectional area of visceral fat, subcutaneous fat, and skeletal muscle.^15^ We quantified its cross-sectional area using standard Huntsfield units (HU). Based on Previous research shows that visceral fat is −150 to −50 HU, subcutaneous fat is −190 to −30 HU, and skeletal muscle is −29 to 150 HU.^11^ The distribution of abdominal fat and skeletal muscle tissue was explored by calculating relevant indicators such as VSR and SMI. A high VSR indicates the accumulation of more visceral adipose tissue, called visceral obesity. Conversely, a lower SMI suggests low muscle mass [Figure 1]. The specific formulas for calculation are as follows^16^:

VSR = area of visceral fat (cm^2^)/area of subcutaneous fat (cm^2^)

SMI = cross-sectional area of the skeletal muscle (cm^2^)/height^2^ (m^2^)

### Statistical analysis

IBM SPSS Statistics version 24 was used for statistical analysis. Analysis of variance (ANOVA) was used to analyze the clinicopathological characteristics of the patients. X-tile 3.6.1 (https://medicine.yale.edu/lab/rimm/research/software.aspx) was used to determine the cut-off point based on minimum *P* values from log-rank χ^2^ statistics for fat-related parameters. Categorical variables were compared using Pearson chi-square or Fisher exact test. Continuous variables were analyzed using the *t*-test or Mann–Whitney *U* test. The Kaplan–Meier method was used to calculate the actual ratio, and group survival rates were compared using the logarithmic rank test. Univariate and multivariate analyses were conducted using Cox proportional hazard regression models. Statistically significant differences were defined at *P* < 0.05.

## Results

### Patient baseline characteristics

Table 1 summarizes the baseline clinical characteristics of 81 patients diagnosed with stage IVB cervical cancer. Among them, 56 (69.1%) were aged <60 years. The patients exhibited a mean BMI of 21.87 ± 3.72 kg/m^2^, a median VSR of 0.60 (0.19, 7.24), and an SMI of 37.29 ± 5.92 cm^2^/m^2^. Of these patients, 64 (79.01%) were diagnosed with squamous cell carcinoma, and 11 (13.58%) had adenocarcinoma. Lymph node metastases were observed in 59 patients (72.84%). All patients underwent radical radiotherapy or chemotherapy, with 40 (49.38%) receiving concurrent chemoradiotherapy.

**Table 1:** Baseline clinical and pathological characteristics of patients.

Characteristics	Total (*N* = 81)
Age (years), *n*(%)	
<60	56 (69.14)
≥60	25 (30.86)
BMI (kg/m^2^), mean ± SD	21.87 ± 3.72
Body composition parameters	
VSR, median [min, max]	0.60 [0.19, 7.24]
SMI (cm^2^/m^2^), mean ± SD	37.29 ± 5.92
VFA (cm^2^), median [min, max]	76.18 [7.10, 256.50]
SFA (cm^2^), median [min, max]	127.00 [2.96, 388.90]
Pathological type, *n* (%)	
Squamous cell carcinomas	64 (79.01)
Adenocarcinoma	11 (13.58)
Other type	6 (7.41)
Differentiation, *n* (%)	
Well	5 (6.17)
Moderately	49 (60.49)
Poorly	27 (33.33)
Lymph node metastasis, *n* (%)	
Yes	59 (72.84)
No	22 (27.16)
Treatment, *n* (%)	
Concurrent chemoradiotherapy	40 (49.38)
Sequential chemoradiotherapy	17 (20.99)
Radiotherapy alone	15 (18.52)
Chemotherapy alone	9 (11.11)

BMI: Body mass index; SFA: Subcutaneous fat area; SMI: Skeletal muscle index; VFA: Visceral fat area; VSR: Visceral-to-subcutaneous adipose tissue area ratio.

**Figure 2:**
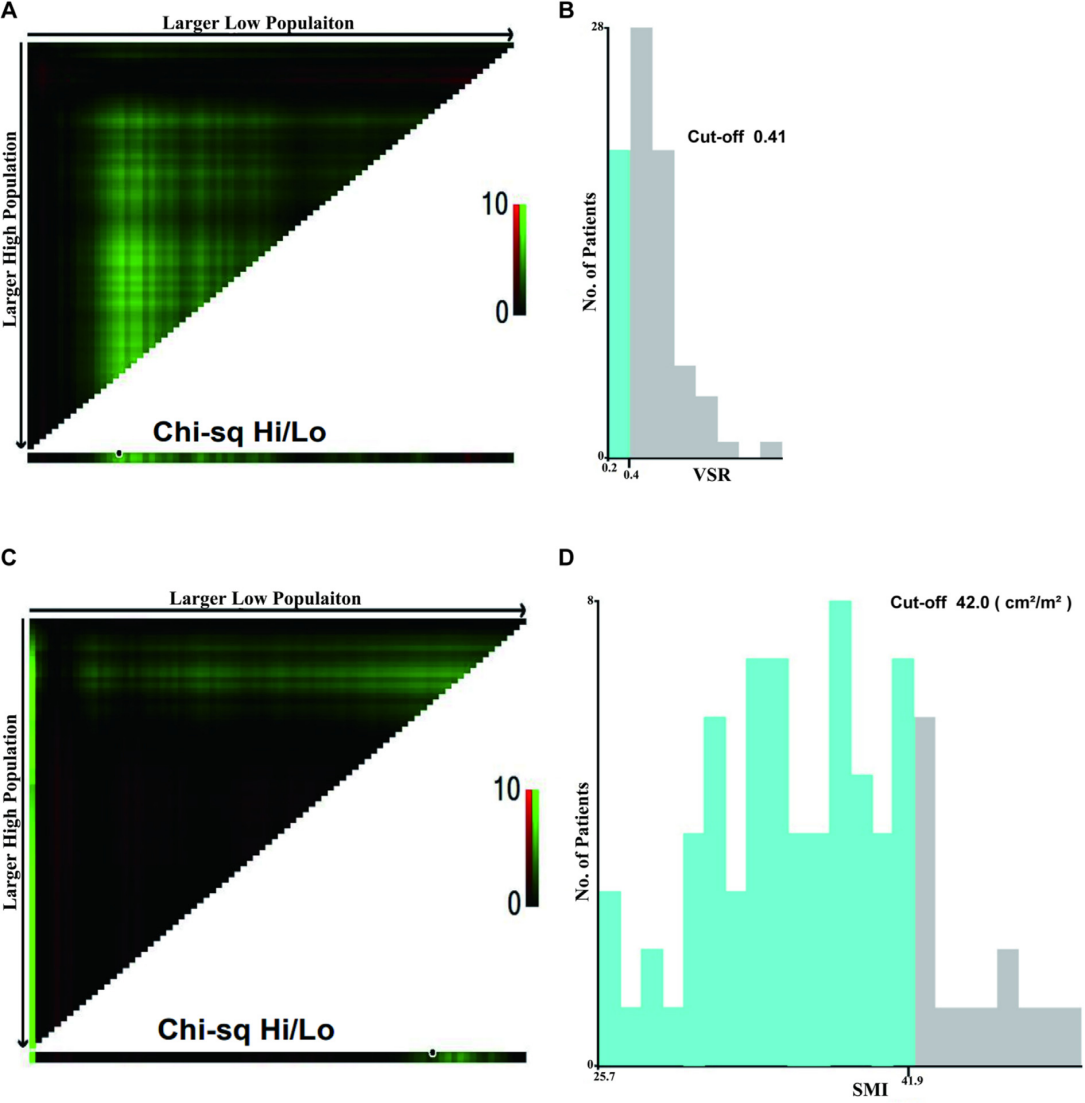
Cut-off points for VSR and SMI determined by the X-tile program. (A–D) X-tile analysis divides the cohort into a training set and a matching validation set based on patient data. Divide the cohort into low and high-count arrays based on the best tangent point. (A and B) the results of VSR and (C and D) the results of SMI. SMI: Skeletal muscle index; VSR: Visceral-to-subcutaneous adipose tissue area ratio.

We determined the cut-off values of VSR and SMI using X-tile analysis [Figure 2]: VSR was set at 0.41, and SMI was set at 42.0 cm^2^/m^2^. Based on the optimal threshold values mentioned above, we categorized patients into high and low visceral obesity groups and high and low muscle mass groups for analysis. We conducted a statistical comparison of the clinical characteristics of the two groups of patients. We found a significant difference in BMI between the high and low VSR groups (*P* = 0.015). In contrast, the two groups had no significant differences in age, pathological type, degree of differentiation, lymph node metastasis, or treatment methods between the two groups [Table 2].

**Table 2:** Clinical characteristics of patients grouped according to VSR and SMI.

Characteristics	VSR-L (*N* = 17)	VSR-H (*N* = 64)	*P* value	SMI-L (*N* = 66)	SMI-H (*N* = 15)	*P* value
Age, *n* (%)			0.105			0.187
<60	15 (88.24)	41 (64.06)		43 (65.15)	13 (86.67)	
≥60	2 (11.76)	23 (35.94)		23 (34.85)	2 (13.33)	
BMI (kg/m^2^), *n* (%)			0.015*			0.143
<24 kg/m^2^	17 (100.00)	48 (75.00)		55 (83.33)	10 (66.67)	
≥24 kg/m^2^	0 (0)	16 (25.00)		11 (16.67)	5 (33.33)	
VSR, *n* (%)			—			0.999
<0.41	—	—		14 (21.21)	3 (20.00)	
≥0.41	—	—		52 (78.79)	12 (80.00)	
SMI (cm^2^/m^2^), *n* (%)			0.999			—
<42	14 (82.35)	52 (81.25)		—	—	
≥42	3 (17.65)	12 (18.75)		—	—	
VFA (cm^2^), *n* (%)			<0.001*			0.544
<100	17 (100.00)	37 (57.81)		45 (68.18)	9 (60.00)	
≥100	0 (0)	27 (42.19)		21 (31.82)	6 (40.00)	
SFA (cm^2^), *n* (%)			0.223			0.680
<100	8 (47.06)	20 (31.25)		24 (36.36)	4 (26.67)	
≥100	9 (52.94)	44 (68.75)		42 (63.64)	11 (73.33)	
Pathological type, *n* (%)			0.401			0.224
Squamous cell carcinomas	12 (70.59)	52 (81.25)		50 (75.76)	14 (93.33)	
Adenocarcinoma	4 (23.53)	7 (10.94)		11 (16.67)	0 (0)	
Other type	1 (5.88)	5 (7.81)		5 (7.57)	1 (6.67)	
Differentiation, *n* (%)			0.099			0.996
Well	0 (0)	5 (7.81)		4 (6.06)	1 (1.23)	
Moderately	14 (82.35)	35 (54.69)		40 (60.61)	9 (11.11)	
Poorly	3 (17.65)	24 (37.50)		22 (33.33)	5 (6.17)	
Lymph node metastasis, *n* (%)			0.943			0.311
Yes	13 (76.47)	46 (71.88)		46 (69.70)	13 (16.05)	
No	4 (23.53)	18 (28.12)		20 (30.30)	2 (2.47)	
Treatment, *n* (%)			0.932			0.376
Concurrent chemoradiotherapy	8 (47.06)	32 (50.00)		30 (45.45)	10 (66.67)	
Sequential chemoradiotherapy	3 (17.65)	14 (21.88)		15 (22.73)	2 (13.33)	
Radiotherapy alone	4 (23.53)	11 (17.19)		14 (21.21)	1 (6.67)	
Chemotherapy alone	2 (11.76)	7 (10.93)		7 (10.61)	2 (13.33)	

*P < 0.05 indicates statistical differences. BMI: Body mass index; SMI: Skeletal muscle index; SMI-H: SMI≥42 cm^2^/m^2^; SMI-L: SMI<42 cm^2^/m^2^; VFA: Visceral fat area; VSR: Visceral-to-subcutaneous adipose tissue area ratio; VSR-H: VSR≥0.41; VSR-L: VSR<0.41;—: No data.

### Survival analysis

**Figure 3:**
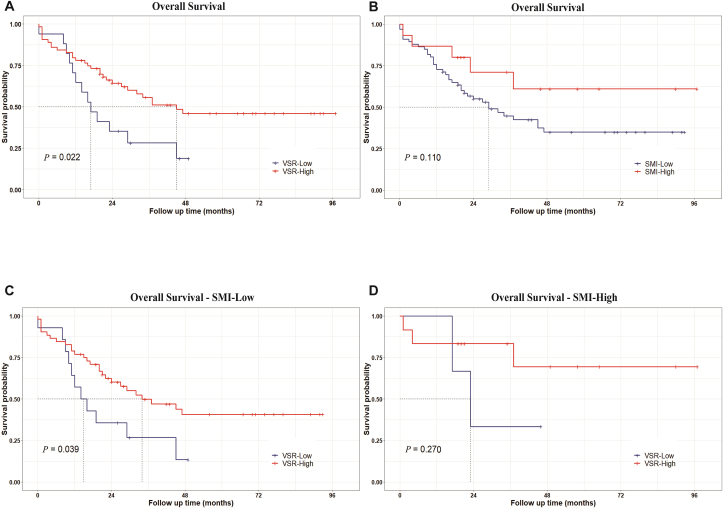
Kaplan–Meier survival analysis of patients grouped according to VSR and SMI. (A) Overall survival rates are classified by visceral obesity and non-visceral obesity. (B) Overall survival rates are classified by sarcopenia and non-sarcopenia. (C) Overall survival rates were classified by visceral obesity and non-visceral obesity in the subgroup of low SMI. (D) Overall survival rates were classified by visceral obesity and non-visceral obesity in the subgroup of high SMI. *P* < 0.05 indicates statistical differences. SMI: Skeletal muscle index; VSR: Visceral-to-subcutaneous adipose tissue area ratio.

We conducted a Kaplan–Meier survival analysis on these 81 patients after VSR and SMI categorization. The high VSR group exhibited significantly better total survival time (overall survival [OS]) than the low VSR group [Figure 3, *P* = 0.022]. In contrast, the two groups had no statistically significant difference in progression-free survival time (PFS). This suggests that patients with visceral obesity have better prognoses after radiotherapy and chemotherapy. After SMI grouping, no significant differences were found in OS and PFS between high- and low-SMI groups, indicating that the impact of skeletal muscle mass on patient prognosis was not significant. Subgroup analysis in the low-SMI group demonstrated a significantly improved OS in the high VSR group than in the low VSR group (*P* = 0.039). Conversely, within the high SMI group, no significant difference in OS existed between the high and low VSR groups (*P* = 0.270). This confirmation underscores the correlation between muscle-wasting visceral obesity and improved patient prognosis following radiotherapy and chemotherapy.

In addition, univariate Cox analysis revealed that low VSR (HR = 2.107, 95% confidence interval [CI] 1.093–4.065, *P* = 0.026) and the use of radiotherapy or chemotherapy alone (HR = 2.044, 95% CI 1.108–3.772, *P* = 0.022) were associated with the prognosis of patients with stage IVB cervical cancer. Furthermore, multivariate Cox analysis was conducted on key factors, indicating that low VSR was an independent risk factor for the prognosis of patients with stage IVB cervical cancer (HR = 1.993, 95% CI 1.008–3.940, *P* = 0.047), suggesting that visceral obesity acts as a protective factor for the prognosis of patients with stage IVB cervical cancer [Table 3].

**Table 3:** Univariate and multivariate Cox regression analysis.

Variables	Univariate analysis		Multivariate analysis	
	Hazard ratio (95% CI)	*P* value	Hazard ratio (95% CI)	*P* value
Age（<60 years）	1.096 (0.563–2.135)	0.787	**—**	**—**
Normal BMI（<24 kg/m^2^）	2.208 (0.869–5.614)	0.096	1.354 (0.500–3.665)	0.551
Low VSR（<0.41）	2.107 (1.093–4.605)	0.026∗	1.993 (1.008–3.940)	0.047∗
Low SMI（<42 cm^2/^m^2^）	2.074 (0.815–5.276)	0.126	1.878 (0.728–4.842)	0.192
VFA (<100 cm^2^)	1.246 (0.639–2.427)	0.518	**—**	**—**
SFA (<100 cm^2^)	1.236 (0.665–2.297)	0.503	**—**	**—**
Squamous cell carcinomas	0.732 (0.369–1.452)	0.372	**—**	**—**
Poorly differentiation	0.833 (0.434–1.597)	0.582	**—**	**—**
No lymph node metastasis	1.176 (0.603–2.290)	0.635	**—**	**—**
Radiotherapy/chemotherapy alone	2.044 (1.108–3.772)	0.022∗	1.861 (0.992–3.494)	0.053

*∗P* < 0.05 indicates statistical differences. BMI: Body mass index; CI: Confidence interval; SFA: Subcutaneous fat area; SMI: Skeletal muscle index; VFA: Visceral fat area; VSR: Visceral-to-subcutaneous adipose tissue area ratio; —: No data.

## Discussion

This retrospective study showed that visceral obesity before treatment was an independent protective factor for the prognosis of patients with stage IV cervical cancer. Furthermore, the OS of patients with visceral obesity was significantly better than that of patients without it. Patients with low skeletal muscle mass before treatment also showed a poor prognosis, although this result was not statistically significant. However, our subsequent subgroup analysis found that the VSR grouping did not affect the prognosis of patients with a high SMI. Among patients with low SMI, those with low VSR had a worse prognosis. We believe these factors increase the risk of death in an additive manner; that is, the prognosis of nonobese patients with muscle loss is very poor. It has been suggested that patients’ general condition and nutritional status affect their prognosis. By focusing on the content and distribution of adipose tissue, we explored and identified reliable markers that could predict the prognosis of patients with stage IVB cervical cancer.

Currently, the preferred treatment for patients with stage IVB cervical cancer involves radiotherapy-based systemic comprehensive treatment, significantly improving patient prognosis. However, previous reports showed that the 5-year survival rate of patients with IVB cervical cancer ranged from 23.7–26.5%, and approximately 50% died due to disease progression within 1 year.^17^ Beyond tumor-specific prognostic factors, assessing patients' functional and nutritional statuses can aid in understanding risk stratification and clinical decision-making for patients with cervical cancer. Many studies have explored the relationship between obesity and mortality in patients with cancer, yet the conclusions remain controversial. Previous studies have shown that VSR is an important prognostic factor for disease-free survival in patients with resectable colorectal cancer. Patients with VSR >0.5 have a shorter disease-free survival.^18^ Park et al. discovered that higher visceral fat (>29% VSR) was associated with better OS in patients with colorectal cancer.^19^ The differences in the above results may be due to different measurement methods, critical values, statistical methods, and distributions of patient characteristics.

Our research demonstrated a relationship between BMI and the VSR index in patients with stage IVB cervical cancer. Considering that individuals with higher BMI tend to exhibit some visceral obesity, we also observed a correlation between higher VSR and better OS. This indicates that visceral obesity is associated with a better prognosis in patients with advanced cervical cancer. There are several possible explanations for the association between visceral obesity and mortality. Energy failure caused by cachexia is more prevalent in patients with cancer than in healthy individuals.^20^ Additionally, visceral adipose tissue contains more inflammatory and immune cells than subcutaneous adipose tissue,^21^ which is conducive to activating immune responses during treatment. Visceral adipose tissue contains glucocorticoids and androgen receptors, which can regulate the body's energy metabolism through hormones affecting Tumor microenvironment (TME) changes. Therefore, high visceral adipose tissue can protect patients from cachexia and synergistically enhance the antitumor response, possibly preventing disease progression and recurrence.

However, the objective assessment of body composition using CT cross-sectional imaging technology can supplement our current clinical and nutritional assessment of patient's health and treatment tolerance. Therefore, nutritional support can be initiated earlier and more appropriately to improve treatment compliance and clinical therapeutic effects.

This study has some limitations. First, this was a retrospective, single-center study, and data collection was based on a review of medical records or follow-up patients. Avoiding inaccurate data recordings or patient recall bias remained difficult despite repeated checks. Second, owing to the small number of patients with stage IVB cervical cancer, we collected as much complete data as possible in the past decade. However, the sample size was still relatively small, which may have caused bias in the results. Finally, our failure to obtain dynamic laboratory test indicators related to disease progression when selecting clinical features may have led to a potential bias. Our findings should be further confirmed in larger prospective cohort studies.

In conclusion, our results confirm the protective effect of visceral obesity before radiotherapy and chemotherapy in patients with stage IVB cervical cancer and the negative prognostic impact of low muscle index and low VFA/SFA ratio. Moreover, this study rarely showed that the proportion of visceral adipose tissue was an important prognostic factor in patients with stage IVB cervical cancer. In addition to tumor-specific prognostic factors, the evaluation of visceral fat and other body composition indicators may help make clinical decisions and improve patients' prognosis and quality of life.

## Funding

The work was supported by the scientific development funding from the First Affiliated Hospital of Xi’an Jiaotong University (No.2020QN-07) and the Fundamental Research Funds for Central Universities (No.xzy012020044).

## Authors contribution

Mengjiao Cai and Jinlu Ma conceived and designed the study; Chao Ji, Silin Liu, Wang, Jie Chen, Jin Wang, and Xinyue Zhang collected and analyzed data. Chao Ji wrote the manuscript. All the authors have read and approved the final manuscript.

## Ethics statement

This retrospective study was conducted in accordance with the *Declaration of Helsinki*, and our research conformed to the privacy rights of human subjects. Patient consent was waived owing to the retrospective study design.

## Data availability statement

The data supporting the findings of this study are available from the corresponding author upon request.

## Conflict of interest

The authors declare that they have no known competing financial interests or personal relationships that could have appeared to influence the work reported in this paper.
